# Lagophthalmos and Frozen Globe as the Initial Presentation of Invasive Breast Carcinoma

**Published:** 2015

**Authors:** Ibrahim Bulent Buttanri, Muslime Akbaba, Didem Serin, Safak Karslioglu, Seyhmus Ari, Selvinaz Ozkara

**Affiliations:** 1 Haydarpaşa Numune Education and Research Hospital, Eye Clinic, Istanbul, Turkey; 2 Istanbul Okuloplasti, Oculoplastic Surgery and Ocular Oncology Center, Istanbul, Turkey; 3 Dicle University, Faculty of Medicine, Department of ophthalmology, Diyarbakir, Turkey; 4 Haydarpaşa Numune Education and Research Hospital, Pathology Clinic, Istanbul, Turkey

**Keywords:** Lagophthalmos, Frozen Globe, Invasive Breast Carcinoma

## Abstract

A 75-year-old woman presented with six months history of progressing lagophthalmos and immobility of the left eye. Magnetic resonance imaging (MRI) of the orbit demonstrated infiltration of orbital fat and the extra-ocular muscles. We performed transverse blepharotomy of the left eyelid to correct lagophthalmos; and during surgery, we took a biopsy sample from levator muscle and orbital fat. After the operation, the patient was able to close her eyelids, and epithelial problems were resolved. Biopsy revealed fibro-vascular, muscle and fat tissue infiltrated with minimally differentiated carcinoma cells. Breast examination revealed a nodule in the left breast. Biopsy of the mass confirmed the diagnosis of invasive breast carcinoma. Orbital manifestation of metastases, such as diplopia, lagophthalmos or pain may reduce life quality of the patients and must be evaluated on a multidisciplinary basis.

## INTRODUCTION

One of the most common cancers to metastasize to the orbit is breast carcinoma (1). Orbital findings generally present three to six years after the diagnosis of a breast carcinoma and orbital metastases as the initial presentations are rare (2-4). This study reports a patient in which lagophthalmos and frozen globe were the initial presentation of breast carcinoma. We performed transverse blepharotomy to correct lagophthalmos, and biopsy to establish the diagnosis.

## CASE PRESENTATION

**Figure 1:**
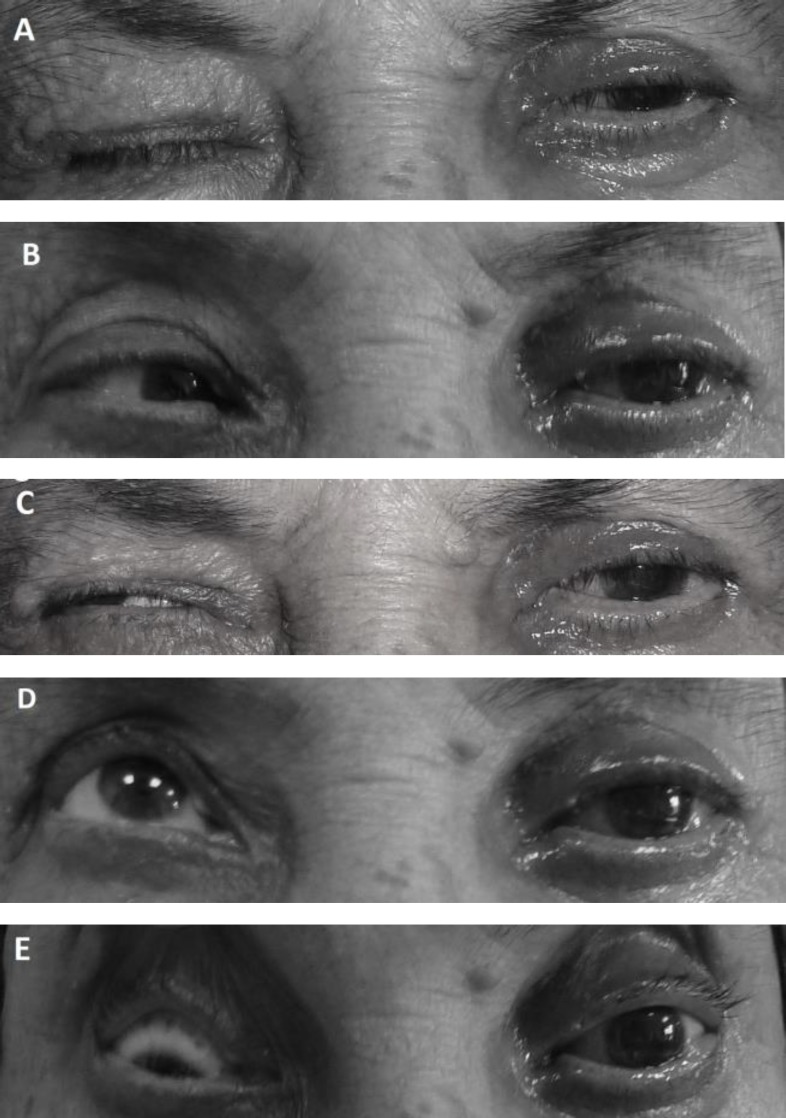
A) The patient presented with lagophthalmos and frozen globe, B) Left gaze, C) Right gaze, D) Upgaze, E) Downgaze

**Figure 2:**
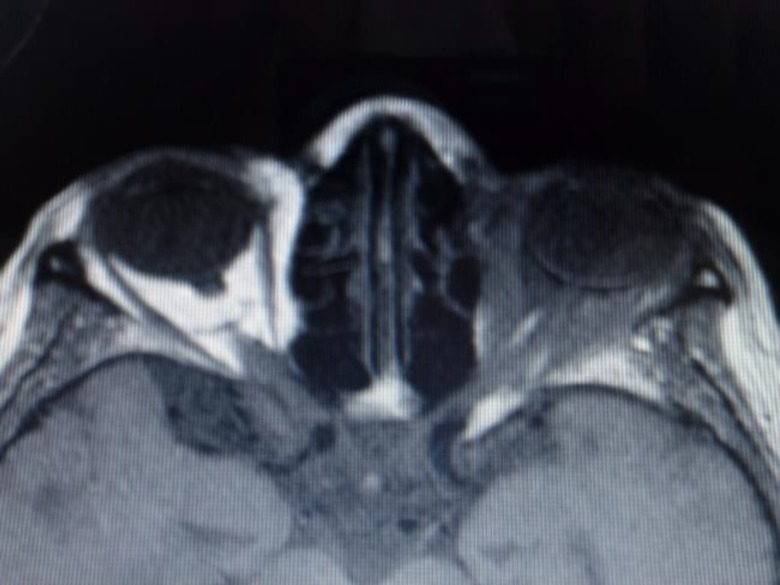
Magnetic Resonance Imaging of the Orbit Demonstrated Infiltration of Orbital Fat and the Extra-Ocular Muscles

A 75-year-old woman presented with six-month history of worsening lagophthalmos (Fig. 1A) and immobility of the left eye (Fig. 1B, 1C, 1D, 1E). In another center, she had undergone steroid treatment with the presumptive diagnosis of ‘idiopathic orbital inflammatory syndrome’ one month before, but her complaints had not resolved. On examination, her best-corrected visual acuity was 20/400. She had epitheliopathy secondary to lagophthalmos. Magnetic resonance imaging (MRI) of the orbit demonstrated infiltration of orbital fat and the extra-ocular muscles (Fig. 2).

**Figure 3:**
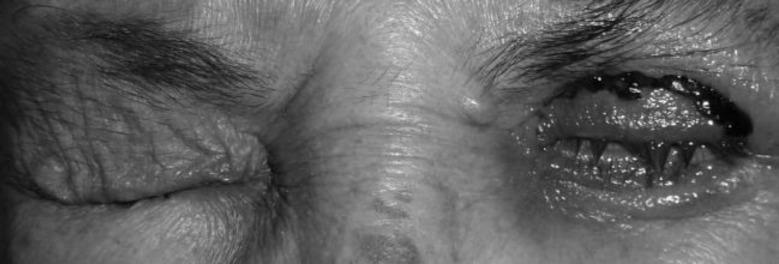
After the Operation, the Patient was Able to Close Her Eyelids and Epithelial Problems were Resolved

**Figure 4:**
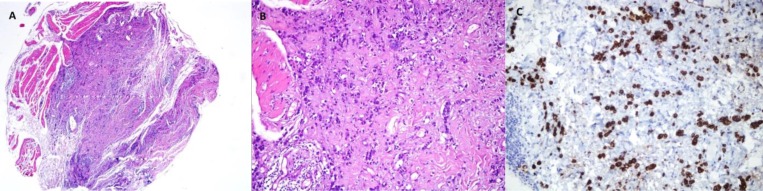
Diffuse infiltration of muscle and fat (H&Ex40), B) Infiltrative cells have large cytoplasms and big hyperchromatic nucleus (H&Ex200), C) Immunhistochemical stain with cytoceratine -7 ANTIBODIES shows uniform strong staining of tumor cells (IHKx200)

We performed transverse blepharotomy of the left eyelid to correct lagophthalmos and during surgery, we performed a biopsy from levator muscle and orbital fat. After the operation, the patient was able to close her eyelids, and epitheliopathy was solved (Fig. 3). Biopsy revealed muscle and fat tissue infiltrated with minimally differentiated carcinoma cells (Fig. 4A-4B). Immunohistochemistry of tumor cells demonstrated staining with estrogen, progesterone, and cytokeratin-7 antibodies (Fig. 4C). The patient was referred to an oncology clinic. Breast examination revealed a nodule in the left breast. Biopsy of the mass confirmed the diagnosis of invasive breast carcinoma.

## DISCUSSION

Orbital metastases may manifest with a palpable mass, proptosis, displacement of the globe, pain, chemosis, inflammation, or eyelid swelling (5) and the patient can be misdiagnosed as idiopathic orbital inflammatory syndrome (6). In patients with orbital inflammation refractory to steroids, biopsy should be considered to make histopathological diagnosis (7). Breast cancer metastases have a tendency to infiltrate the extraocular muscles and the surrounding fat tissue and may cause a motility deficit (8). The lagophthalmos in this patient was due to infiltration of the levator muscle and the other eyelid structures which resulted in restriction and immobility of the upper eyelid. Lagophthalmos has not been reported as the presenting symptom of breast carcinoma before and up to our knowledge, this is the first report.

Ocular metastases can be treated with systemic chemotherapy and hormonal therapy with or without orbital radiation (9). Extensive ocular surgery is not curative and may cause ocular morbidity, therefore not recommended in patients with metastases (9). In our patient, the effect of transverse blepharotomy may not be permanent because of the progressive nature of the disease.

The mean survival time of the patients with orbital metastases is reported to be between 18 and 22 months (3). Orbital complications of metastases such as diplopia, lagophthalmos or pain may reduce life quality of the patients and must be evaluated on a multidisciplinary basis.
